# Temporal trends in occupational mortality in Brazil, 2010-2019

**DOI:** 10.47626/1679-4435-2023-1111

**Published:** 2024-02-16

**Authors:** Ana Lys Marques Feitosa, Déborah Ellen de Matos Ribeiro, Fernando Ferraz do Nascimento, Hilda Maria Martins Bandeira, Márcio Denis Medeiros Mascarenhas, Malvina Thaís Pacheco Rodrigues

**Affiliations:** 1 Programa de Pós-Graduação em Saúde e Comunidade, Universidade Federal do Piauí, Teresina, PI, Brazil

**Keywords:** occupational mortality, time series studies, health information systems, mortalidade por acidentes de trabalho, estudos de séries temporais, sistemas de informação em saúde

## Abstract

**Introduction:**

Occupational accidents represent a severe and complex public health issue.

**Objectives:**

To identify temporal trends in occupational mortality in Brazil from 2010 to 2019.

**Methods:**

This was an ecological study with time series analysis using data from the Brazilian Ministry of Health Mortality Information System (Ministério da Saúde/ Sistema de Informações sobre Mortalidade). The mortality rate was calculated using Prais-Winsten estimation.

**Results:**

In the study period, 34,683 work-related deaths were recorded in Brazil, with a higher occurrence among White (51.0%) men (94.3%) aged 20 to 39 years (44.8%). The highest proportion of deaths (16.5%) was identified in the state of São Paulo. Regarding sex, temporal trends were stable for both men (annual percentage change [APC] = -1.7; 95%CI -3.9 to 0.7) and women (APC = -0.8; 95%CI -1.8 to 0.2). The age groups up to 19 years (APC = -5.1; 95%CI -9.0 to l.l) and 20 to 39 years (APC = -3.3; 95%CI -6.0 to -0.5) showed a decreasing trend, while the remaining age groups showed a stable trend. Black race had a decreasing trend (APC = -8.1; 95%CI -10.7 to -5.5), while White (APC = -2.3; 95%CI -38.0 to 0.2) and mixed races (APC = -1.2; 95%CI -5.2 to 2.9) had a stable trend. Eight states showed a decreasing trend; only the state of Pará (APC = 2.1; 95%CI 0.8 to 3.4) showed an increasing trend, while the other states had a stable trend.

**Conclusions:**

Temporal trends in occupational mortality were stable for most of the indicators evaluated. There is a lack of measures contributing to occupational safety and health in Brazil.

## Abstract (pt)

**Introdução:**

Acidentes de trabalho representam um grave e complexo problema de saúde pública. Objetivos: Analisar a tendência temporal da mortalidade por acidentes de trabalho no Brasil no período de 2010 a 2019.

**Métodos:**

Estudo ecológico de séries temporais, com dados do Sistema de Informações sobre Mortalidade. Calculou-se a taxa de mortalidade utilizando regressão de Prais-Winsten.

**Resultados:**

Foram registrados 34.683 óbitos decorrentes de acidentes de trabalho no país, com maior ocorrência no sexo masculino (94,3%), na faixa etária de 20 a 39 anos (44,8%) e na raça branca (51,0%). A maior proporção de óbitos foi identificada no estado de São Paulo (16,5%). A tendência temporal apresentou estabilidade no sexo masculino (variação percentual anual (VPA) = -1,7; IC95% -3,9 a 0,7) e feminino (VPA = -0,8; IC95% -1,8 a 0,2). A tendência revelou decréscimo nas faixas etárias de até 19 anos (VPA = -5,1; IC95% -9,0 a 1,1) e de 20 a 39 anos (VPA = -3,3; IC95% -6,0 a -0,5), enquanto as demais faixas apresentaram estabilidade. A raça preta apresentou decréscimo (VPA = -8,1; IC95% -10,7 a -5,5), enquanto a branca (VPA = -2,3; IC95% -38,0 a 0,2) e a parda (VPA = -1,2; IC95% -5,2 a 2,9) apresentaram estabilidade. Oito unidades da federação apresentaram decréscimo; apenas o estado do Pará (VPA = 2,1; IC95% 0,8 a 3,4) apresentou acréscimo, enquanto as demais unidades registraram estabilidade.

**Conclusões:**

A tendência temporal da taxa de mortalidade apresentou estabilidade na maior parte dos indicadores avaliados. Percebe-se a carência de discussões que possam contribuir com ações no campo da segurança e saúde no trabalho.

## INTRODUCTION

Occupational accidents (OAs) represent a severe and complex public health issue. The International Labour Organization estimates that, since 2012, over 21,000 workers have died due to OAs or occupational diseases in Brazil. Among the countries in the Americas and those comprising the G20, a group formed by the world's major economies, Brazil ranks second globally in deaths due to OAs, following only Mexico.^1^

OA is defined as a sudden and severe event occurring in the course of work activities. Its notification to public and private health services is mandatory, regardless of the worker's employment or social security status. OAs cause potential or immediate harm to the employee's health and can cause bodily injury or functional impairment that directly or indirectly leads to death, loss, or permanent or temporary reduction in the functional capacity for work.^2^

The association of death with work is recorded in the death certificate under the field 'Work-related accident,' within the section on 'External causes'. It aims to specify the circumstances of non-natural death, particularly whether the event triggering the death was work-related.^3,4^

Considering the aforementioned and the complexity surrounding OAs, this study aimed to identify temporal trends in occupational mortality in Brazil from 2010 to 2019 to determine a behavioral pattern over time.

## METHODS

This is an ecological study with time series analysis using data from the Brazilian Ministry of Health (Ministério da Saúde) Mortality Information System (Sistema de Informações sobre Mortalidade - SIM), available at the IT Department of the Brazilian Unified Health System (Departamento de Informática do Sistema Único de Saúde do Ministério da Saúde - DATASUS) website. Only deaths in which the field 'Work-related accident' was marked 'yes' were selected for analysis.

Population estimates by municipality, age, and sex reported by the Brazilian Ministry of Health based on data from the Brazilian Institute of Geography and Statistics (Instituto Brasileiro de Geografia e Estatística - IBGE) were used for calculating the mortality rate. The following variables were analyzed: a) sex (male or female); b) age groups in years (up to 19; 20 to 39; 40 to 59; and 60 years or older); c) race/color (White, Black, and mixed race) - data available from 2012 onwards; and d) Brazilian state.

Mortality rates were calculated by dividing the number of work-related deaths by the total population residing in the state and multiplying the result by 100,000 inhabitants. Temporal trends in occupational mortality were identified, and their correlation with sex, age group, race/color, and Brazilian state were determined from 2010 to 2019 (data on race were only available from 2012 onwards).

Data were retrieved from the DATASUS website, entered into a Microsoft Excel spreadsheet, and analyzed using Prais-Winsten estimation, which takes into account the present value of a time series using previous values in time in a linear regression model, on Stata version 14.0 (StataCorp LP, College Station, USA). Annual percent change (APC) values and their respective 95%CIs were obtained. The trend was considered increasing if beta was positive and p < 0.05, decreasing if beta was negative and p < 0.05, and stable if p > 0.05.^5^

According to Resolution no. 466/2012 of the Brazilian National Health Council, this study was exempt from research ethics committee approval for using publicly available data and not exposing the participant's personal information.

## RESULTS

From 2010 to 2019, 34,683 work-related deaths were recorded in Brazil. Most deaths occurred in White (51.0%) men (94.3%) aged to 20 to 39 years (44.8%) (Table 1). The state of São Paulo registered the highest proportion of deaths (16.5%), followed by the states of Minas Gerais (12.5%) and Paraná (10.7%) (Table 3).

**Table 1.** Work-related deaths by sex, age group, and race in Brazil, 2010-2019

Variables	n	%
Sex		
Male	32704	94.3
Female	1,965	5.7
Total	34,683	100.0
Age group (years)		
Up to 19	1,396	4.0
20 to 39	15.540	44.8
40 a 59	13,743	39.6
60 or more	3,954	11.4
Total	34,683	100.0
Race*		
White	17273	51.0
Black	2,068	6.1
Mixed	14,501	42.8
Total†	33,842	100.0

Source: Brazilian Ministry of Health Mortality Information System (Ministério da Saúde/ Sistema de Informações sobre Mortalidade - SIM). * Data on race available from 2012 onwards. † Also includes Yellow, Indigenous, and not declared.

**Table 3.** Work-related deaths by Brazilian state, 2010-2019

State	n	%
Acre	167	05
Alagoas	132	0.4
Amapá	103	0.3
Amazonas	683	2.0
Bahia	1750	5.0
Ceará	634	1.8
Federal District	311	0.9
Espírito Santo	734	2.1
Goiás	1476	4.3
Maranhão	976	2.8
Mato Grosso	1823	5.3
Mato Grosso do Sul	736	2.1
Minas Gerais	4,330	12.5
Pará	1581	4.6
Paraíba	224	0.6
Paraná	3,710	10.7
Pernambuco	726	2.1
Piauí	545	1.6
Rio de Janeiro	1,034	3.0
Rio Grande do Norte	231	0.7
Rio Grande do Sul	2,654	7.7
Rondônia	856	2.5
Roraima	107	0.3
Santa Catarina	2,629	7.6
São Paulo	5,707	16.5
Sergipe	300	0.9
Tocantins	524	1.5
Total	34,683	100.0

Source: Brazilian Ministry of Health Mortality Information System (Ministério da Saúde/ Sistema de Informações sobre Mortalidade - SIM).

Occupational mortality trends by sex were stable for both male (APC = -1.7; 95%CI -3.9 to 0.7) and female sex (APC = -0.8; 95%CI -1.8 to 0.2). The age groups up to 19 years (APC = -5.1; 95%CI -9.0 to 1.1) and 20 to 39 years (APC = -3.3; 95%CI -6.0 to -0.5) showed a decreasing trend, while the other age groups showed a stable trend. Regarding race, the Black race exhibited a decreasing trend (APC = -8.1; 95%CI -10.7 to -5.5), while the White (APC = -2.3; 95%CI -38.0 to 0.2) and mixed races (APC = -1.2; 95%CI -5.2 to 2.9) had a stable trend.

Of 27 states, 8 showed a decreasing temporal trend: the Federal District (APC = -15.1; 95%CI -20.1 to -9.7), Maranhão (APC = -4,6; 95%CI -6.2 to -2.9), Mato Grosso (APC = -3.6; 95%CI -6.4 to -0.7), Mato Grosso do Sul (APC = -6.3; 95%CI -9.8 to -2.6), Paraná (APC = -3.1; 95%CI -5.1 to -1.2), Pernambuco (APC = -5.9; 95%CI -9.8 to -1.9), Rio de Janeiro (APC = -4.4; 95%CI -5.9 to -3.0), and São Paulo (APC = -6.2; 95%CI -8.0 to -4.3). Only Pará (APC = 2.1; 95%CI 0.8 to 3.4) showed an increasing temporal trend, while the other states had a stable trend (Table 4).

**Table 4.** Trends in the occupational mortality rate (per 100,000 habitants) by Brazilian state, 2010-2019

State	Mortality rate		APC (%)	95%CI	p-value	Trend
	2010	2019				
Acre	0.65	2.04	10.0	-3.8 to 25.8	0.139	Stable
Alagoas	0.56	0.33	-7.8	-16.6 to 1.9	0.098	Stable
Amapá	1.58	1.89	1.0	-13.2 to 17.6	0.879	Stable
Amazonas	1.60	1.47	-1.6	-8.0 to 5.1	0.579	Stable
Bahia	1.10	1.10	-1.3	-5.6 to 3.2	0.515	Stable
Ceará	0.78	0.84	1.4	-3.0 to 6.1	0.479	Stable
Federal District	3.37	0.43	-15.1	-20.1 to -9.7	<0.001	Decreasing
Espírito Santo	1.97	1.47	-3.9	-9.0 to 1.4	0.127	Stable
Goiás	2.44	1.89	-1.8	-5.1 to 1.5	0.243	Stable
Maranhão	1.55	1.00	-4.6	-6.2 to -2.9	<0.001	Decreasing
Mato Grosso	5.63	5.19	-3.6	-6.4 to -0.7	0.022	Decreasing
Mato Grosso do Sul	3.05	2.12	-6.3	-9.8 to -2.6	0.004	Decreasing
Minas Gerais	1.75	3.07	3.1	-2.7 to 9.2	0.261	Stable
Pará	1.87	2.20	2.1	0.8 to 3.4	0.006	Increasing
Paraíba	0.60	0.42	-1.6	-5.8 to 2.7	0.398	Stable
Paraná	3.44	2.78	-3.1	-5.1 to -1.2	0.006	Decreasing
Pernambuco	1.04	0.56	-5.9	-9.8 to -1.9	0.009	Decreasing
Piauí	1.63	1.40	-1.9	-4.7 to 0.9	0.161	Stable
Rio de Janeiro	0.74	0.50	-4.4	-5,9 to -3,0	<0.001	Decreasing
Rio Grande do Norte	0.56	0.71	-1.4	-7.9 to 5.4	0.630	Stable
Rio Grande do Sul	2.25	2.48	0.2	-1.2 to 1.7	0.715	Stable
Rondônia	3.88	5.46	3.3	-0.1 to 6.8	0.056	Stable
Roraima	1.96	2.31	-0.7	-3.8 to 2.6	0.642	Stable
Santa Catarina	3.86	4.02	-1.1	-4.0 to 1.9	0.426	Stable
Sao Paulo	1.65	1.04	-6.2	-8.0 to -4.3	<0.001	Decreasing
Sergipe	1.80	1.30	-5.3	-11.9 to 1.8	0.120	Stable
Tocantins	4.17	3.18	-2.2	-6.5 to 2.1	0.268	Stable
Total	1.80	1.66	-1.5	-4.0 to 1.0	0.205	Stable

Source: Brazilian Ministry of Health Mortality Information System (Ministério da Saúde/ Sistema de Informações sobre Mortalidade - SIM). APC = annual percentage change.

## DISCUSSION

OAs generate various problems not only for the worker but also for the company and the state. Even in less severe cases, employees receive paid sick leave, causing the employer to lose a part of their workforce and having to shoulder the costs associated with days missed. Severe OAs entail costs for the state, as they require prolonged absence from the worker, as well as expenses for the health care system. The National Social Security Institute (INSS) is responsible for providing benefits such as paid sick leave, disability retirement, and survivors benefits.^6^

Occupational mortality is associated with poor working conditions and the ineffectiveness of workplace regulations.^7^ In Brazil, the risk of work-related death was significantly higher among White men aged 20 to 39 years. This could be explained by this ethnic group constituting the largest portion of the formally employed population in the country.

Sex and age group findings are associated with the characteristics of the working population, as well as with jobs that involve greater risks and physical stress, as the worker must have a physical size appropriate to the role.^8^ Temporal trends by sex remained stable throughout the historical series. However, the age groups up to 19 years and from 20 to 39 years showed a decreasing temporal trend. The remaining age groups showed a stable trend.

Contrary to our findings, an estimation of the temporal trend of typical OAs specifically in the textile and clothing industry in the state of Santa Catarina, from 2008 to 2017, revealed a significant decrease in the risk of OAs (8.8%).^9^ Although the primary focus of this study was to assess temporal trends in occupational mortality, it is worth noting the scarcity of other studies on this topic for comparative analysis of the data.

As for the characterization of fatal OAs in Brazil, in Minas Gerais and in the municipality of Uberaba, from 1997 to 2006, there was a prevalence of men aged 25 to 44 years.^10^ Regarding fatal OAs resulting from urban violence in Campinas, São Paulo, of the 82 reported cases, 74 (90.2%) were men, with a mean age of 42 years and a median of 43 years.^11^

Of the 114 fatal OAs identified in Palmas, Tocantins, between 2007 and 2015, almost all occurred in men (93.8%) aged 30 to 49 years (46.5%).^12^ This scenario, in addition to being related to the age group most actively engaged in work, indicates the demographic most exposed to risks in the work environment.

The Southeast region, which encompasses states such as São Paulo and Minas Gerais, had the highest proportion of deaths. This is attributed to its higher population density, making it the most economically active region in the country, holding industrial, commercial, and financial relevance. This region also has a higher number of employment relationships and more activities in the commerce, transportation, construction, and water and sewage sectors. Moreover, the Southeast region is monitored more easily and rigorously by labor inspectors, reducing informal labor and contributing to an increased notification of accidents compared with other regions.^6^

Another factor to consider is urban violence in major cities such as São Paulo, which also promotes commuting accidents—occurring between home and the place of work—and road transportation accidents, reflecting in the high number of OAs.^13^

Analyzing the data in light of the National Policy on Occupational Health, established by Ordinance no. 1,823 of August 23, 2012, it should be emphasized that the policy should be applied to all workers, prioritizing individuals and groups in situations of vulnerability, particularly those engaged in informal and precarious activities.^14^

In trying to provide equitable care for workers, there is a need to ensure that working conditions are supervised, with the aim of preventing accidents and safeguarding groups exposed to precarious conditions. By determining the epidemiological characteristics of work-related deaths, it becomes possible to formulate effective strategies targeted at the most vulnerable populations.

Another point worth mentioning concerns underreporting and the poor quality of information. The analysis of various sources of records of work-related deaths in Brazilian information systems indicated extensive underreporting, demonstrating that work-related deaths are underestimated and that despite improvements over time, the quality of reporting is still poor.^12,15^ Therefore, a limitation of this study includes the potential insufficiency of data in records and underreporting of cases, meaning that caution should be taken when interpreting these results.

Identifying the characteristics of OAs is an important tool for the implementation of measures to reduce occupational risks and improve working conditions. This enables preventive actions against occupational hazards, providing insights for both employees and employers to identify risks at an early stage.^16^

## CONCLUSIONS

In addition to recognizing the need for increased accuracy when reporting OAs, our analysis of occupational mortality from 2010 to 2019 in Brazil identified stable temporal trends for most of the indicators analyzed. Furthermore, there is a noticeable lack of discussions and actions in the field of occupational health and safety. Based on the results of this study, measures should be designed and implemented to reduce OAs and intensify preventive measures for workers' health through awareness and supervision.

## Figures and Tables

**Table 2.** Occupational mortality trends (per 100,000 habitants) by sex, age group, and race in Brazil, 2010-2019

Variables	Mortality rate		APC (%)	95%CI	p-value	Trend
	2010	2019				
Sex						
Male	3.46	3.16	-1.7	-3.9 to 0.7	0.140	Stable
Female	0.20	0.22	-0.8	-1.8 to 0.2	0.091	Stable
Total	0.00	0.00	34.1	-38.8 to 193.9	0.414	Stable
Age group (years)						
Up to 19	0.23	0.17	-5.1	-9.0 to -1.1	0.020	Decreasing
20 to 39	2.67	2.12	-3.3	-6.0 to -0.5	0.026	Decreasing
40 to 59	2.91	2.78	-1.6	-3.4 to 0.2	0.075	Stable
60 or more	1.61	1.65	0.2	-2.0 to 2.5	0.852	Stable
Total	1.80	1.66	-1.6	-3.8 to 0.7	0.152	Stable
Race	2012*	2019				
White	2.10	1.86	-2.3	-38.0 to 0.2	0.064	Stable
Black	1.65	1.03	-8.1	-10.7 to-5.5	<0.001	Decreasing
Mixed race	1.62	1.58	-1.2	-5.2 to 2.9	0.487	Stable
Total^†^	2.84	2.59	-1.6	-3.6 to 0.3	0.088	Stable

Source: Brazilian Ministry of Health Mortality Information System (Ministério da Saúde/ Sistema de Informações sobre Mortalidade - SIM). APC = annual percentage change. * Data on race available from 2012 onwards. ^†^ Also includes Yellow, Indigenous, and not declared.
